# Stability-Guaranteed and High Terrain Adaptability Static Gait for Quadruped Robots

**DOI:** 10.3390/s20174911

**Published:** 2020-08-31

**Authors:** Qian Hao, Zhaoba Wang, Junzheng Wang, Guangrong Chen

**Affiliations:** 1 School of Information and Communication Engineering, North University of China, Taiyuan 030051, China; diah123hao@nuc.edu.cn (Q.H.); wangzb@nuc.edu.cn (Z.W.); 2 State Key Laboratory of Intelligent Control and Decision of Complex Systems, School of Automation, Beijing Institute of Technology, Beijing 100081, China; wangjz@bit.edu.cn; 3 Robotics Research Center, Beijing Jiaotong University, Beijing 100044, China

**Keywords:** high terrain adaptability, quadruped robots, static gait, stability, rough terrains

## Abstract

Stability is a prerequisite for legged robots to execute tasks and traverse rough terrains. To guarantee the stability of quadruped locomotion and improve the terrain adaptability of quadruped robots, a stability-guaranteed and high terrain adaptability static gait for quadruped robots is addressed. Firstly, three chosen stability-guaranteed static gaits: intermittent gait 1&2 and coordinated gait are investigated. In addition, then the static gait: intermittent gait 1, which is with the biggest stability margin, is chosen to do a further research about quadruped robots walking on rough terrains. Secondly, a position/force based impedance control is employed to achieve a compliant behavior of quadruped robots on rough terrains. Thirdly, an exploratory gait planning method on uneven terrains with touch sensing and an attitude-position adjustment strategy with terrain estimation are proposed to improve the terrain adaptability of quadruped robots. Finally, the proposed methods are validated by simulations.

## 1. Introduction

Nowadays, legged robots have been a research hotspot and gained various attention [1,2,3,4]. Quadruped robots have the advantages of fast motion speed, high flexibility, strong terrain adaptability, and high stability [5], which results in their great application prospects in material transportation, engineering exploration, rescue, and military investigation. Therefore, the research of quadruped robot has an important theoretical significance and a practical value. In addition, there are many famous quadruped robots in the world nowadays, such as Bigdog [6], HyQ [7], HyQ2MAX [8], StarlETH [9], Anymal [10], MIT Cheetah 1∼3 [11,12,13], Cheetah-cub [14], minitaur [15], and AlienGo [16], etc.

Quadruped robot gait consists of static gait [17] and dynamic gait [18]. In simple sum, static gait is slow, but it is more stable and it has a high terrain adaptability [19], while dynamic gait is faster, but its stability is poor and its terrain adaptability is relatively low [20].

Dynamic quadruped robots are hard to control because they are nonlinear, unstable, time-variant, and multi-input multi-output (MIMO) systems. They also interact with environment and behave switch dynamics, especially during support exchange [21]. Research group in Italian Institute of Technology (IIT) proposed a feedback/feedforward control structure using inverse dynamics approach in HyQ [22]. Boston Dynamics employed the idea of spring loaded inverted pendulum (SLIP) model to guide the locomotion of Bigdog [23]. In addition, a hierarchical controller [11,24] was utilized to do the quadrupedal locomotion in Anymal [10] and MIT Cheetah [12,14].

As for static gait, Nie et al. employed some optimization methods to do the motion planning algorithm of static walking gait for a quadruped robot [25]. Focchi et al. proposed a planning/control framework for quasi-static walking of quadrupedal robots to tackle high slope terrains [26]. Li et al. studied a hierarchically planning static gait for quadruped robot walking on rough terrain [27]. Wilshin et al. investigated the longitudinal quasi-static stability predicts changes in dog gait on rough terrain [28]. Luo et al. proposed a method based on the body lateral adjustment to improve the static gait performance of a quadruped robot [29]. Zhang et al. studied a continuous static gait with twisting trunk of a metamorphic quadruped robot [30]. Gorner et al. presented a 3D odometry algorithm for statically stable walking robots that only uses proprioceptive data delivered by joint angle and joint torque sensors embedded within the legs [31]. Zhang et al. investigated a static gait planning method based on terrain complexity estimation without any machine vision system for quadruped robot walking on unknown rough terrain [32]. As thus, quadruped robots have the ability to walk on different terrains with stability-guaranteed via motion planning, body adjustment, and self-perception.

Stability is a prerequisite for legged robots to execute tasks and traverse rough terrains. The legged robot is expected to be an environment-accessible platform because of its environmental adaptability. With the consideration of stability, walking speed, and energy consumption or Cost of Transport (CoT) [33], the quadruped walking robot will be one of the most practical locomotion machines to move above on uneven terrain [34]. In addition, it will be the stablest while walking in a static state [35]. According to the nature of the stability, studies of quadruped gaits can be divided in two parts: static stability and dynamic stability. Note that static stability can be named ’positional stability’ as an ability to remain stable in position with respect to all other influencing dynamics, especially when intertwined with other control mechanism. Static stability assumes that the vertical projection of the center of gravity (CoG) always remains inside the stability polygon with an adequate stability margin during all phases of movements. The stability margin ensures that, whatever speed the robot reaches, it will not be carried away by its own momentum, and consequently tip over and fall down. Therefore, static walking gait is a better choice walking on complex terrains. However, there are many kinds of complex terrains. Even though the static walking gait is relatively mature, the research about how to control the quadruped robot traverse over a high platform is still less. Moreover, the environmental adaptability issue of quadruped robot without environmental perception system should be addressed further as well.

Meanwhile, compliance control is an effective way to reduce body oscillations and maintain the stability of robot. In order to improve the environmental compliance of a quadruped robot on rough terrain, impedance control [36,37] has been studied to reduce the contact impact, which would be too large in high precision position control [38,39]. Impedance control can be divided into position-based impedance control [40] and force-based impedance control [41]. Referring to [42,43], a position/force based impedance control is employed to achieve the compliant behavior of a quadruped robot on rough terrain in this paper.

In this paper, the overall aim is to look for a static gait of quadruped robots with the biggest stability margin and its control strategy for high terrain adaptability. Firstly, the static gait of quadruped robots with the biggest stability margin is chosen by comparing three classical stability-guaranteed static gaits: intermittent gait 1&2 and coordinated gait. Secondly, a position/force based impedance control is employed to achieve the compliant behavior of quadruped robots on rough terrain, which will maintain the stability of robot well. Thirdly, an exploratory gait planning method on uneven terrains with touch sensing and an attitude-position adjustment strategy with terrain estimation are investigated to further improve the terrain adaptability of quadruped robots. This research is meaningful for quadruped robots, especially when considering the carriage of heavy goods [17].

The rest of this paper is organized as follows: Section 2 describes the system model of research subject: a quadruped robot. Section 3 investigates three chosen stability-guaranteed static gaits and chooses the static gait with the biggest stability margin to do a further research. Section 4 studies an exploratory gait planning on uneven terrain with touch sensing and an attitude-position adjustment strategy to improve the terrain adaptability of quadruped robots. Section 5 presents the simulation results to validate the proposed methods. Simulations includes comparison of three chosen static gaits, walk on platform, and walk on uneven terrain. Section 6 draws the conclusions.

## 2. System Model of the Quadruped Robot

**Figure 1:**
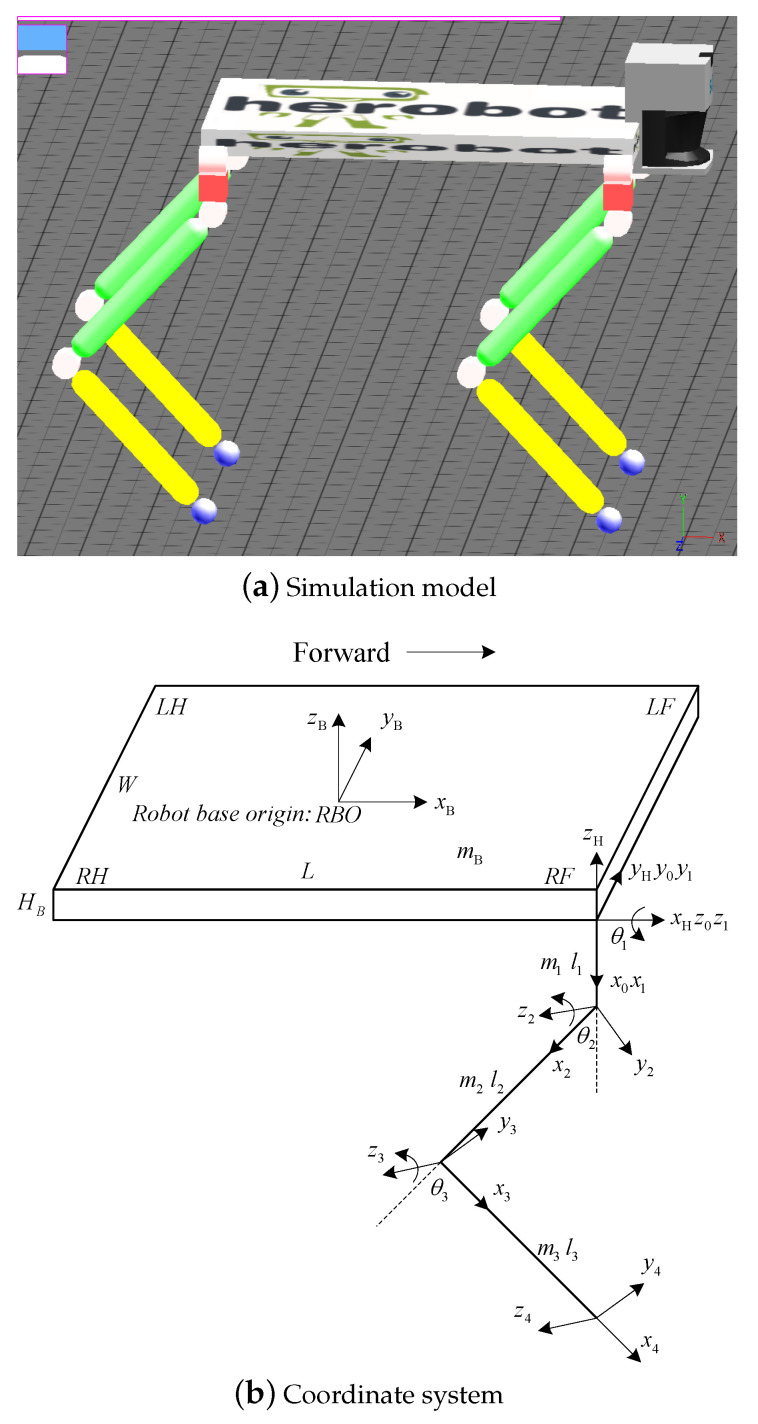
The simulation model and coordinate system of the quadruped robot.

The simulation model setup by WEBOTS software is shown in Figure 1a and the coordinate system of quadruped robot is shown in Figure 1b. Note that this paper addresses a high terrain adaptability static gait for a quadruped robot without using the equipped lidar and camera in Figure 1a. There are three degrees of freedom (DoF) in each leg: 2 DoFs in hip joint (roll & pitch) and 1 DoF in knee joint (pitch). The front and hind legs are both elbow style, and the same Jacobian matrix can be obtained as follows:

(1) $$J=\left[\begin{array}{ccc} 0 & l_{2}c_{2}+l_{3}c_{23} & l_{3}c_{23}\\ l_{1}c_{1}+l_{2}c_{1}c_{2}+l_{3}c_{1}c_{23} & -l_{2}s_{1}s_{2}-l_{3}s_{1}s_{23} & -l_{3}s_{1}s_{23}\\ l_{1}s_{1}+l_{2}s_{1}c_{2}+l_{3}s_{1}c_{23} & l_{2}c_{1}s_{2}+l_{3}c_{1}s_{23} & l_{3}c_{1}s_{23} \end{array}\right]$$

where $s_{i},c_{i}$ are $\sin\theta_{i},\cos\theta_{i}$, respectively. $s_{ij},c_{ij}$ are $\sin\left(\theta_{i}+\theta_{j}\right),\cos\left(\theta_{i}+\theta_{j}\right)$, respectively.

The DH (Denavit–Hartenberg) method [44,45,46] can be applied to derive robot kinematics as follows:

(2) $${}^{B}p_{\mathrm{FOOT}}={\left[\begin{array}{ccc} x & y & z \end{array}\right]}^{T}=\mathrm{FK}\left(q\right)$$

(3) $$q={\left[\begin{array}{ccc} \theta_{1} & \theta_{2} & \theta_{3} \end{array}\right]}^{T}=\mathrm{IK}\left({}^{B}p_{\mathrm{FOOT}}\right)$$

where ${}^{B}p_{\mathrm{FOOT}}$ is the coordinates of foot under body coordinate system, and FK and IK are forward kinematics and inverse kinematics, respectively.

The Lagrange method can be applied to derive robot dynamics as follows:

(4) $$\begin{array}{c} Q={\left[\begin{array}{ccc} \ddot{q} & \dot{q} & q \end{array}\right]}^{T}=\mathrm{FD}\left(\tau \right) \end{array}$$

(5) $$\begin{array}{c} \tau ={\left[\begin{array}{ccc} \tau_{1} & \tau_{2} & \tau_{3} \end{array}\right]}^{T}=\mathrm{ID}\left(Q\right) \end{array}$$

where FD and ID are forward dynamics and inverse dynamics, respectively.

## 3. Three Stability-Guaranteed Static Gaits

### 3.1. Workspace

**Figure 2:**
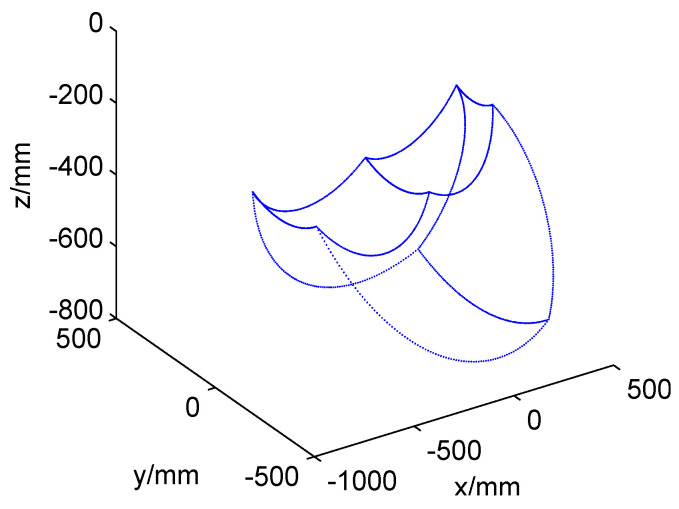
The workspace of the right fore leg of quadruped robot (boundary lines are drawn).

The workspace of foot end-effector of robot can be defined as the collection of all positions that can be reached in space by the foot end-effector under the hip joint coordinate system {H}. It is mainly determined by the motion range of joint angle. The workspace of the right fore leg is shown in Figure 2.

It can be seen that the workspace is an irregular three-dimensional space, and it is difficult to get its analytic expression. However, gait planning must be carried out in the workspace, and the point outside the workspace is where the robot can not reach. In order to simplify the analysis, a simpler method based on the restriction of workspace boundary is adopted since the workspace boundary of the robot is determined by the angle range of each joint. It is decided that, once the difference between the planned joint angle and the corresponding upper or lower limit is less than a certain threshold $\theta_{m}$ and continues to decrease, stop gait planning to decrease the difference or plan the gait to increase the difference. It yields

(6) $$\begin{array}{c} \left\{\begin{array}{c} \left|\theta -\theta_{\min}\right|<\theta_{m}\\ \dot{\theta }<0 \end{array}\right.or\left\{\begin{array}{c} \left|\theta -\theta_{\max}\right|<\theta_{m}\\ \dot{\theta }>0 \end{array}\right. \end{array}$$

where θ is the joint angle, and $\theta_{\max},\;\theta_{\min}$ are the upper and lower limit of joint angle, respectively. Only when the joint angle is within its allowable range of motion can the robot’s own parts not interfere with each other.

### 3.2. Stability Margin

**Figure 3:**
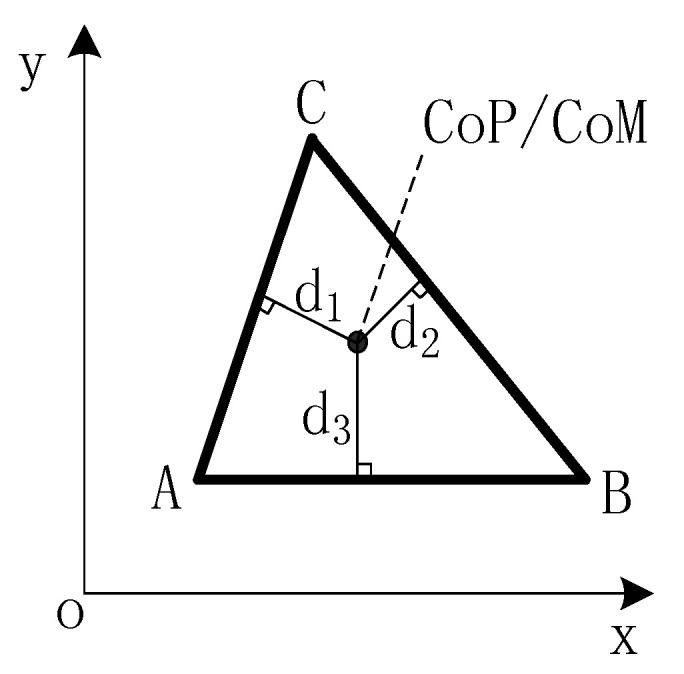
Stability margin of quadruped robot: the shortest distance between the CoP/CoM of robot and each side of the polygon.

Zero moment point (ZMP) is a popular stability criterion for legged robots [47]. For simplicity, center of pressure (CoP) is used to replace ZMP to do stability-guaranteed gait planning. The basis of steady static walking is that CoP of robot on the ground always falls in the polygon area formed by the end-effectors of supporting legs. Stability margin is a quantitative index of static walking stability. It refers to the shortest distance between the CoP of robot and each side of the polygon, as shown in Figure 3.

The formula for calculating CoP is

(7) $$\begin{array}{c} x_{CoP}=\frac{F_{Az}x_{A}+F_{Bz}x_{B}+F_{Cz}x_{C}}{mg}\\ y_{CoP}=\frac{F_{Az}y_{A}+F_{Bz}y_{B}+F_{Cz}y_{C}}{mg} \end{array}$$

where *m* is the mass of robot, $A(x_{A},y_{A})$, $B(x_{B},y_{B})$, $C(x_{C},y_{C})$ are the coordinates of supporting leg end-effectors. The calculation of CoP needs to use the force in the vertical direction of foot end-effector of supporting legs. For simple, the projection of center of mass (CoM) on the support surface can be utilized to replace CoP and CoM can be arranged on the geometric center of robot trunk. Then, the stability margin is

(8) $$SM=\min\left\{d_{1},d_{2},d_{3}\right\}$$

where $d_{1},d_{2},d_{3}$ are the distances from the projection of the CoM to each side of the support polygon.

**Remark** **1.** *In static walking, the projections of robot base origin (RBO), CoM, CoP, and ZMP could be equivalent to the same point on the ground by setting proper counterweights, even though there is a little bit difference among them. As such, through planning and controlling the projection of RBO inside the support polygon area formed by the end-effectors of supporting legs, the static walking stability can be guaranteed. If the projection of RBO falls in the inner center of support triangle, the static walking has the highest stability.*

### 3.3. Three Stability-Guaranteed Static Gaits

Static gait of the quadruped robot can be divided into two classical gaits: intermittent gait and coordinated gait. Intermittent gait refers to the separation of leg swinging and CoM movement; coordinated gait refers to the simultaneous movement of swinging leg and CoM.

**Figure 4:**
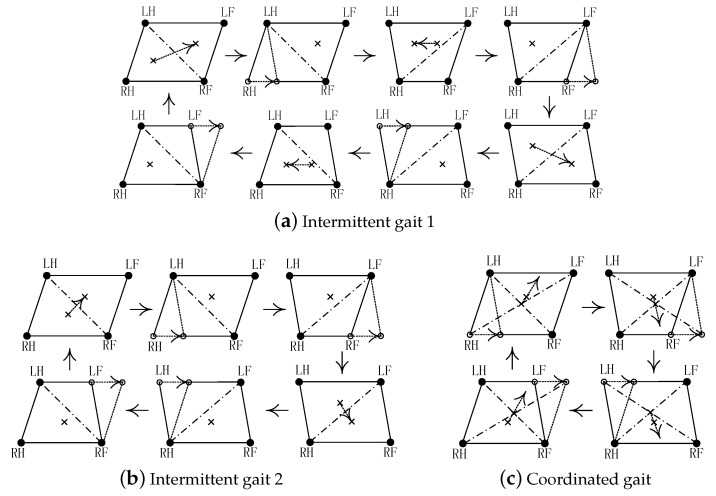
Three typical stability-guaranteed static gaits.

Referring to the static walking of quadruped animals and based on the stability margin, gait naturalness and movement space requirements, the basic static walking rules are given as: (1) Hind leg moves firstly between legs on the left or right side in the same row; (2) Backward leg moves firstly between legs on the fore or hind side in the same column; (3) Each movement of CoM follows the walking forward direction as far as possible. Under these rules, three typical stability-guaranteed static gaits investigated in this paper are given: (1) In intermittent gait 1, as shown in Figure 4a, the robot moves its CoM once every step; (2) In intermittent gait 2, as shown in Figure 4b, the robot moves its CoM once every two steps; (3) In coordinated gait, as shown in Figure 4c, the robot moves its CoM and step leg synchronously and simultaneously.

The three typical stability-guaranteed static gaits are shown as rotate clockwise for one step cycle in Figure 4. Assuming that each step length of robot is *S* and the time of each action (take a step or move CoM once) is *T*, the gait cycles of three static gaits are 8T,6T,4T, respectively. However, the traveling distance of robot *S* is the same in a gait cycle. Thus, the walking speeds of three typical static gaits are S/8T,S/6T,S/4T, respectively. Support leg and swing leg move at the same time in coordinated gait, and then the robot can walk faster, smoother, and bionic. However, its projection point of CoM almost adjacent to the boundary of support polygon so that its stability margin is the lowest and robot would fall easily. Intermittent gait 1 has the greatest stability margin if its CoM trajectory follows the inner center of support triangle ($d_{1}=d_{2}=d_{3}$) at each step. However, its CoM movement will occur opposite movement when comparing with walking forward direction, which results in redundant CoM movement, time and energy waste, and walking speed decrease. Intermittent gait 2 is a compromise between the above two gaits. Its stability margin is higher than that of coordinated gait, and its walking speed is higher than that of intermittence gait 1.

In summary, the increase of walking speed will inevitably lead to the decrease, even the disappearance of stability margin. In order to seek a greater increase in walking speed, robot is usually forced to switch from static gait to dynamic gait. In addition, the stability margin is replaced by dynamic stability in dynamic balance control, which is not discussed in this paper. Taking stability as a prerequisite, the following research focuses on intermittent gait 1. In Section 5, the relationship among walking speed, energy consumption, and stability of the three static gaits will be further analyzed.

## 4. High Terrain Adaptability

High terrain adaptability of quadruped robot can be achieved by two ways: adaptive gait planning on uneven terrain and attitude-position adjustment.

### 4.1. Gait Planning

#### 4.1.1. Gait Planning on Even Terrain

Reasonable gait planning will reduce the contact impact when foot end-effectors of robot touch down on the ground. Gait planning includes support phase and swing phase. In this paper, the cycloid trajectory planning method is used to plan support phase gait as

(9) $$\begin{array}{c} q=q_{0}+\left(q_{d}-q_{0}\right)\frac{\varphi -\sin\varphi }{2\pi }\\ \varphi =\frac{2\pi t}{T_{sp}} \end{array}$$

where $q={\left[\begin{array}{cccccc} x & y & z & \alpha & \beta & \gamma \end{array}\right]}^{T}$ are the position and attitude of robot. $q_{0}$ and $q_{d}$ are the initial and desired point in gait planning. $T_{sp}$ is the time of support phase. The CoM trajectory is planned like the cross in Figure 4. Combining the coordinates of each support leg in body coordinate system {B}, each joint trajectory can be obtained via Equation (3).

The swing phase gait is planned by the polynomial interpolation trajectory method [47], and it yields

(10) $$f\left(t\right)=a_{0}+a_{1}t+\cdots +a_{n}t^{n}$$

where n+1 is the number of constraints in gait planning. According to the principle of zero impact [47], there are four constraints to make sure that the instantaneous velocity and acceleration of touching down and lifting off are all zero. Adding to two constraints for initial and final position, and the other n−5 custom constraints, then the planned trajectory of swing phase can be calculated.

#### 4.1.2. Exploratory Gait Planning on Uneven Terrain with Touch Sensing

**Figure 5:**
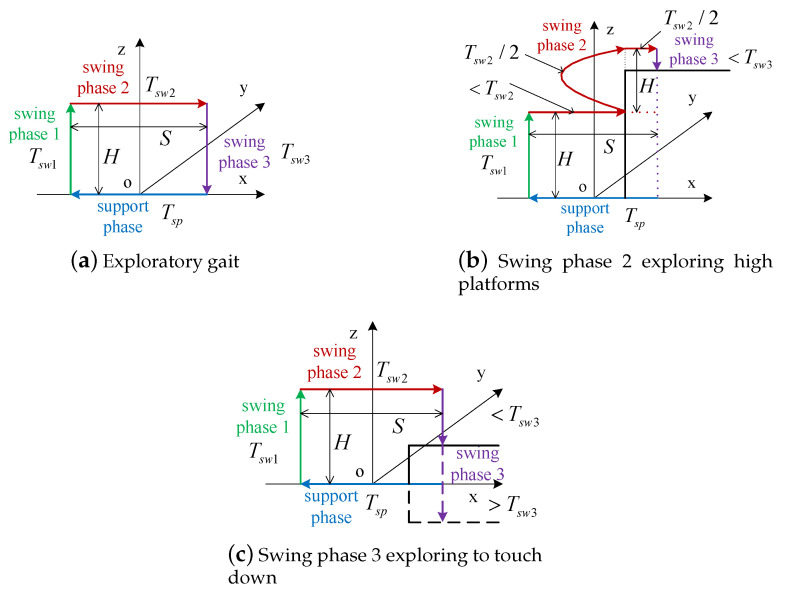
Principle of exploratory gait.

Because of the oscillations of robot trunk, the contact impact of foot end-effector still will occur on the even terrain with the gait planning under principle of zero impact. Even through impedance control can reduce the impact efficiently, the impact is not small enough to ensure the stable walking on uneven terrain, such as stairs, slopes, gravel and high platforms, etc. The walking stability is always at the first place for legged robots. Therefore, intermittent gait 1 is adopted to do the gait planning on complex terrains. In addition, an exploratory gait planning on uneven terrain with touch sensing is proposed as shown in Figure 5. Exploratory gait incorporates force sensing [48] on the foot, and it is a planning method in which a robot can adjust gait planning actively when its foot meets obstacles during swing phase. Four three-dimensional force sensors are installed at the foot end-effectors of quadruped robot to achieve the proposed touch sensing.

Exploratory gait is divided into four parts: support phase and swing phase 1,2,&3. The cycloid trajectory planning method Equation (9) is employed on the four parts as well. The planning time of support phase and swing phase are $T_{sp}$ and $T_{sw}$, respectively. As shown in Figure 5, it is with low probability to touch high platforms or touch down in swing phase 1, but it is with high probability to touch high platforms in swing phase 2 and touch down in swing phase 3. To try to avoid oscillations and reduce the contact impact, the planning time of swing phase 1,2,&3 are set as $1/5T_{sw},2/5T_{sw},2/5T_{sw}$, respectively. More time for the swing motion will cause a lower speed of a foot end-effector and result in lower contact impact.

The decomposition of the swing phase makes the gait own the exploratory ability/function in swing phases 2&3. The swing phase 1 raises the foot by *H*. The swing phase 2 can explore high platforms as shown in Figure 5b. When the foot is subjected to a force in the reverse direction along the *x* axis and the force is greater than the given threshold, it is determined that there is a high platform in the front. At the moment, the swing leg retracts and returns to its original position in the horizontal direction and raises upward by H/2 in the vertical direction. To avoid the limit of workspace, the CoM is raised vertically by H/2 by support legs at the same time. After the foot position is raised again by *H* (H/2+H/2), the foot continues to reach forward to complete the unfinished step. Then, it turns to the swing phase 3 to finish touching down the ground, and the swing phase ends. If the foot encounters a high platform again, repeat the previous action until the workspace is limited, and it is determined that the high platform can not be crossed (obstacles).

Swing phase 3 can explore touching down, as shown in Figure 5c. When the foot is subjected to force along the direction of the *z*-axis and the force greater than the given threshold, the touching down of the foot is determined and the swing phase is terminated. If the foot is still not touching down the ground after probing *H* depth, then it continues probing at the speed of $H/T_{sw}$ until touching down the ground to complete the swing phase. If the foot beyond the workspace without touching down, it is determined that the gap cannot be crossed.

The advantages of proposed exploratory gait planning method are as follows: (1) Touching down sensing is added, and it can deal with complex terrain calmly and enhance mobility; (2) Gait planning is relatively simple and parameters S,H are not affected by terrain; (3) The strong walking stability of intermittent gait 1 is inherited.

### 4.2. Position/Force Based Active Compliance Control

**Figure 6:**
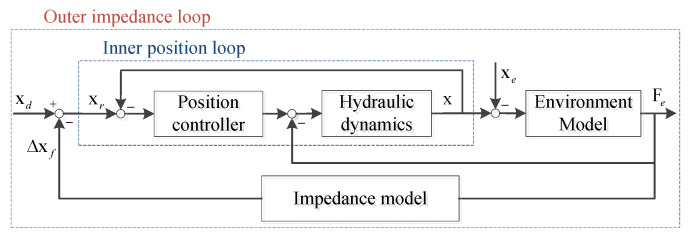
The diagram of position/force based active compliance controller.

To handle the problem of friendly environmental interaction, a position/force based active compliance controller in our previous work [49], as shown in Figure 6. In the diagram of position/force based active compliance controller, only a high performance position control is required without considering an accurate dynamics model of robot and a force tracking control. As thus, the proposed compliance controller is easier to implement in practical use and more reliable for hydraulic actuated robot.

Generally, a second-order linear system (spring-damping-inertia system) is adopted as the desired impedance [42,43]

(11) $$Z_{f}\left(s\right)=\frac{\Delta x_{f}\left(s\right)}{F_{e}\left(s\right)}=\frac{1}{Ms^{2}+Ds+K}$$

The detailed model and stability analysis can be found in [49].

### 4.3. Attitude-Position Adjustment Strategy

The environmental perception sensors, such as lidar and camera, are not reliable and do not work well in complex environments. They also may be broken sometimes. Therefore, the attitude–position adjustment strategy without the environmental perception system should be addressed [50].

After the swing phase, the CoM moves to continue completing *S* step length in the support phase. On the premise of walking stability of a robot, it is necessary to adjust the position and pose of robot according to the terrain for reserving more workspace for the next leg stride. The environment perception system composed of lidar and a camera is a direct way to detect the front terrain, but it can not accurately detect the terrain under the foot and body in real time. There is a big deviation in the estimation by combining moving speed of robot and environmental sensing data. Sometimes, the robot is without an environment perception system or its environment perception system is broken. Therefore, it is necessary to estimate the current terrain by combining robot attitude sensor and foot position.

**Figure 7:**
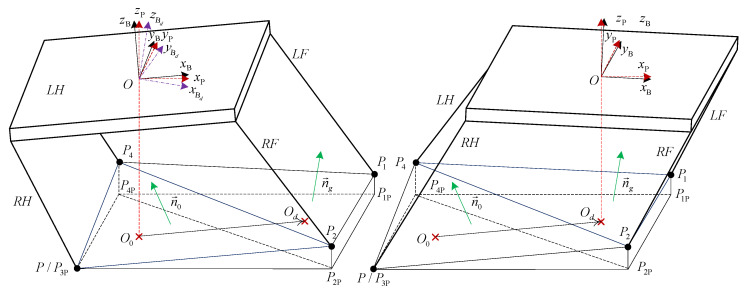
Attitude-position adjustment strategy.

Equation (9) is the expression of attitude-position adjustment, including CoM position adjustment (x,y,z) and robot trunk attitude adjustment (α,β,γ, roll, pitch, yaw). Take intermittent gait 1 into consideration, and assume that the robot has finished LF leg stride and begins to move CoM to stride RH leg. As shown in Figure 7, where {B}, {P} and {$B_{d}$} are the body, horizontal, and desired body coordinate systems, respectively. $P_{1},P_{2},P_{3},P_{4}$ and $P_{1P},P_{2P},P_{3P},P_{4P}$ are the footholds and their projections on the horizontal plane, respectively. *O* is the CoM position, $\Delta P_{2}P_{3}P_{4}$ and $\Delta P_{1}P_{2}P_{4}$ are the current support surface and the support surface in the next leg stride, respectively. ${\vec{n}}_{0}$ and ${\vec{n}}_{g}$ are the normal vectors of $\Delta P_{2}P_{3}P_{4}$ and $\Delta P_{1}P_{2}P_{4}$, respectively. $O_{0}$ and $O_{d}$ are the projections of current and desired CoM position on the horizontal plane $P_{1P}P_{2P}P_{3P}P_{4P}$.

#### 4.3.1. Terrain Estimation

The terrain can be estimated by the direction angle of normal vector of support surface in the next leg stride of robot. The formula for calculating the normal vector ${\vec{n}}_{g}$ can be written as follows:

(12) $${\vec{n}}_{g}=\left[\begin{array}{ccc} x_{g} & y_{g} & z_{g} \end{array}\right]=\vec{P_{1}P_{2}}\times \vec{P_{2}P_{4}}$$

Then, its direction angle can be written as

(13) $$\alpha_{g}=-\arctan\frac{y_{g}}{\sqrt{x_{g}^{2}+z_{g}^{2}}},\beta_{g}=\arctan\frac{x_{g}}{z_{g}}$$

Equation (13) is the estimated terrain parameters.

#### 4.3.2. Attitude Adjustment Strategy

Define α,β,γ as rotation angles of XYZ axes of robot body coordinate system {B}, respectively. The positive direction of rotation is selected according to the right-hand rule, and the rotation order is Z−Y−X. The coordinates of four foot end-effectors under coordinate system {B} and coordinate system {P} with the same origin are $P_{i}^{B}$ and $P_{i}^{P},i=1,2,3,4$, respectively. Their coordinate transformation formula is

(14) $$P_{i}^{B}=T\left(\alpha \right)T\left(\beta \right)T\left(\gamma \right)P_{i}^{P}$$

where

(15) $$\begin{array}{c} T\left(\alpha \right)=\left[\begin{array}{ccc} 1 & 0 & 0\\ 0 & \cos\alpha & \sin\alpha \\ 0 & -\sin\alpha & \cos\alpha \end{array}\right],\\ T\left(\beta \right)=\left[\begin{array}{ccc} \cos\beta & 0 & -\sin\beta \\ 0 & 1 & 0\\ \sin\beta & 0 & \cos\beta \end{array}\right],\\ T\left(\gamma \right)=\left[\begin{array}{ccc} \cos\gamma & \sin\gamma & 0\\ -\sin\gamma & \cos\gamma & 0\\ 0 & 0 & 1 \end{array}\right] \end{array}$$

are all orthogonal matrices and satisfy $T^{T}\left(•\right)=T\left(-•\right)=T^{-1}\left(•\right)$. Then, Equation (14) can be written as

(16) $$P_{i}^{P}=T\left(-\gamma \right)T\left(-\beta \right)T\left(-\alpha \right)P_{i}^{B}$$

Assume the attitude angles of a desired coordinate system {$B_{d}$} are $\alpha_{d},\beta_{d},\gamma_{d}$, which can be given based on Equation (13) without setting yaw or keeping yaw unchanged ($\gamma_{d}=\gamma_{0}$, $\gamma_{0}$ is the initial yaw angle). Then, the foot end-effectors under the desired coordinate system {$B_{d}$} are

(17) $$\begin{array}{cc} P_{i}^{B_{d}} & =T\left(\alpha_{d}\right)T\left(\beta_{d}\right)T\left(\gamma_{d}\right)P_{i}^{P}\\ \; & =T\left(\alpha_{d}\right)T\left(\beta_{d}\right)T\left(\gamma_{d}\right)T\left(-\gamma \right)T\left(-\beta \right)T\left(-\alpha \right)P_{i}^{B} \end{array}$$

Getting the foot end-effectors $P_{i}^{B_{d}}$ under a desired coordinate system and planning their trajectories $P_{i}^{B}\to P_{i}^{B_{d}}$, the required attitude adjustment $\left(\alpha ,\beta ,\gamma \right)\to \left(\alpha_{d},\beta_{d},\gamma_{d}\right)$ can be achieved. Referring to the cycloid trajectory planning method Equation (9), it yields

(18) $$\begin{array}{cc} \; & P_{i}^{B_{d}}\left(t\right)\\ = & T\left(\alpha +\lambda \left(\alpha_{d}-\alpha \right)\right)T\left(\beta +\lambda \left(\beta_{d}-\beta \right)\right)\\ \; & \cdot T\left(\gamma +\lambda \left(\gamma_{d}-\gamma \right)\right)T\left(-\gamma \right)T\left(-\beta \right)T\left(-\alpha \right)P_{i}^{B}\\ \lambda & =\frac{\varphi -\sin\varphi }{2\pi },\varphi =\frac{2\pi t}{T} \end{array}$$

where *T* is the time of attitude adjustment. When t=0, $P_{i}^{B_{d}}\left(0\right)=P_{i}^{B}$; when t=T, $P_{i}^{B_{d}}\left(T\right)=P_{i}^{B_{d}}$. Then, smooth and continuous attitude adjustment is realized.

Keeping the yaw angle unchanged ($\gamma_{d}=\gamma_{0}$), in order to get an optimal desired attitude angle $\alpha_{d},\beta_{d},\gamma_{d}$ that considers four factors: stability margin, CoT, obstacle surmounting ability/workspace, uniform torque distribution, and trails of quadruped robot walking on different terrains are implemented. Combining the maximum slope that the robot can walk up and down and through curve fitting, the adjustment strategy of attitude angles $\alpha_{d},\beta_{d},\gamma_{d}$ can be obtained as

(19) $$\begin{array}{c} \beta_{d}=k_{\alpha \beta }\beta_{g}\\ \alpha_{d}=k_{\alpha \beta }\alpha_{g}\\ k_{\alpha \beta }=\left\{\begin{array}{c} \frac{\beta_{g}}{\beta_{\max}},\beta_{g}\geq 0\\ \frac{\beta_{g}}{\beta_{\min}},\beta_{g}<0 \end{array}\right. \end{array}$$

where the maximum slope that robot can walk up and down are $\beta_{\max}=0.6$ rad and $\beta_{\min}=-0.4$ rad, respectively. $k_{\alpha \beta }$ is a factor that terrain affects attitude.

#### 4.3.3. Position Adjustment Strategy

In order to obtain the maximum of stability margin, the CoM position adjustment strategy is employed to make the projection of CoM on the centroid of next supporting surface/polygon as far as possible. Then, $O_{0}$ and $O_{d}$ should be the inner centers of triangle $\Delta P_{2P}P_{3P}P_{4P}$ and $\Delta P_{1P}P_{2P}P_{4P}$, respectively. It is easy to obtain the coordinates of $O_{0}$ and $O_{d}$ under body coordinate system {B}, which are $x_{O_{0}^{B}},y_{O_{0}^{B}},z_{O_{0}^{B}}$ and $x_{O_{d}^{B}},y_{O_{d}^{B}},z_{O_{d}^{B}}$, respectively.

Since Equation (9) is in the world coordinate system, to facilitate the analysis in the body coordinate system, it can be converted into adjusting the change of CoM Δx,Δy,Δz. It is easy to know the change in horizontal level of CoM, which is as follows:

(20) $$\Delta x=x_{O_{d}^{B}}-x_{O_{0}^{B}},\Delta y=y_{O_{d}^{B}}-y_{O_{0}^{B}}$$

As shown in Figure 7, the robot just finishes LF leg stride. Assuming that the initial CoM height of robot is $H_{0}$, the CoM height should satisfy the following condition to ensure enough workspace for the next leg stride:

(21) $$H_{0}=\frac{\left|z_{P_{2}^{B}}+z_{P_{3}^{B}}+z_{P_{4}^{B}}\right|}{3}$$

Combining Equation (17), it yields the change of CoM in the vertical direction as follows:

(22) $$\Delta z=H_{0}-\frac{\left|z_{P_{2}^{B_{d}}}+z_{P_{3}^{B_{d}}}+z_{P_{4}^{B_{d}}}\right|}{3}$$

The change of foot end-effectors can be deduced from the change of CoM. Then, the same gait planning as Equation (18) can be employed. Furthermore, the attitude and position adjustment strategy can be implemented simultaneously by summing them; this yields

(23) $$\begin{array}{cc} \; & P_{i}^{B_{d}}\left(t\right)\\ = & T\left(\alpha +\lambda \left(\alpha_{d}-\alpha \right)\right)T\left(\beta +\lambda \left(\beta_{d}-\beta \right)\right)\\ \; & \cdot T\left(\gamma +\lambda \left(\gamma_{d}-\gamma \right)\right)T\left(-\gamma \right)T\left(-\beta \right)T\left(-\alpha \right)P_{i}^{B}\\ \; & -\lambda {\left[\begin{array}{ccc} \Delta x & \Delta y & \Delta z \end{array}\right]}^{T} \end{array}$$

In order to further improve the traveling speed, this paper adopts the average speed planning method. Assuming that the given average velocity of attitude adjustment and CoM movement are $V_{a}$ and $V_{d}$, respectively, the action durations are respectively

(24) $$\begin{array}{cc} T_{a} & =\frac{\max\left\{\left|\alpha_{d}-\alpha \right|,\left|\beta_{d}-\beta \right|,\left|\gamma_{d}-\gamma \right|\right\}}{V_{a}},\\ T_{d} & =\frac{\max\left\{\left|\Delta x\right|,\left|\Delta y\right|,\left|\Delta z\right|\right\}}{V_{d}} \end{array}$$

Then, the planning trajectory can be obtained via Equation (9). The swing phase can be planned in the same way.

## 5. Simulations

**Figure 8:**
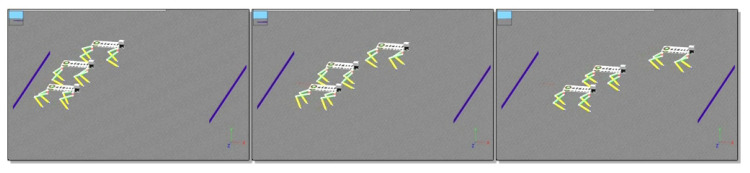
Simulations of quadruped robot walking with three static gaits.

**Figure 9:**
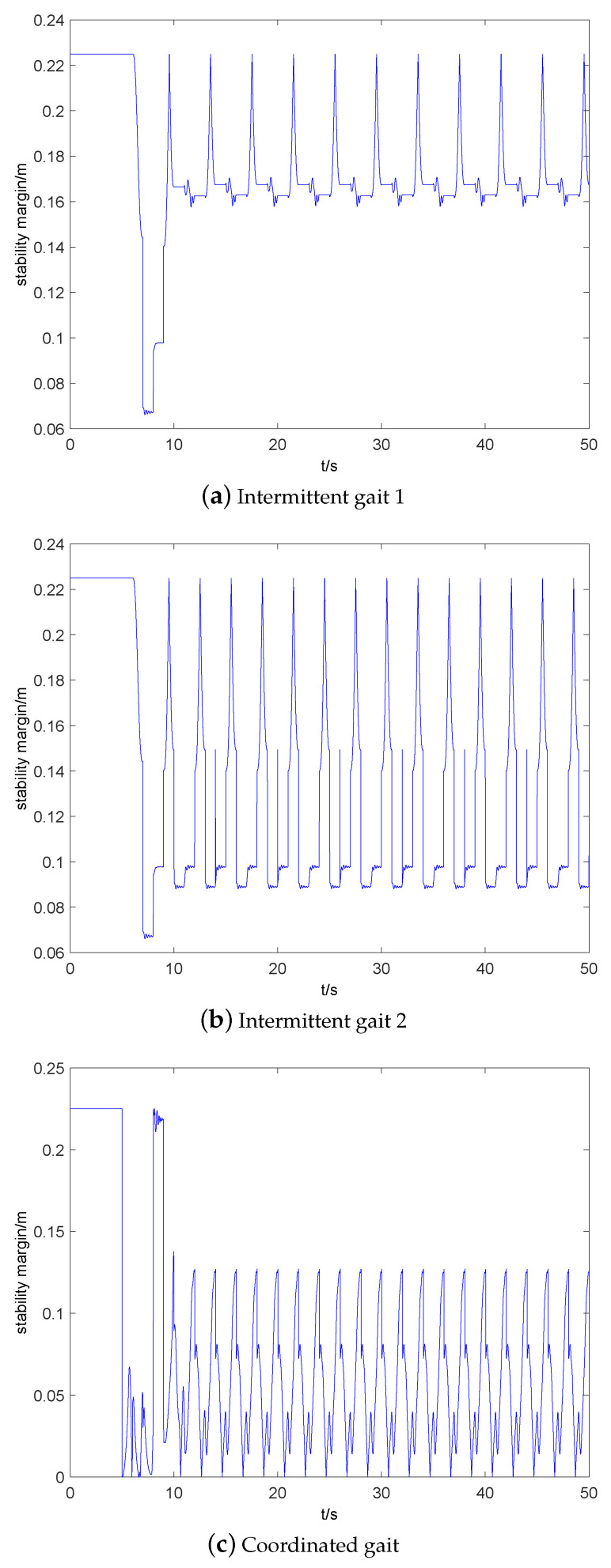
Comparison of the stability margin of three static gaits.

**Figure 10:**
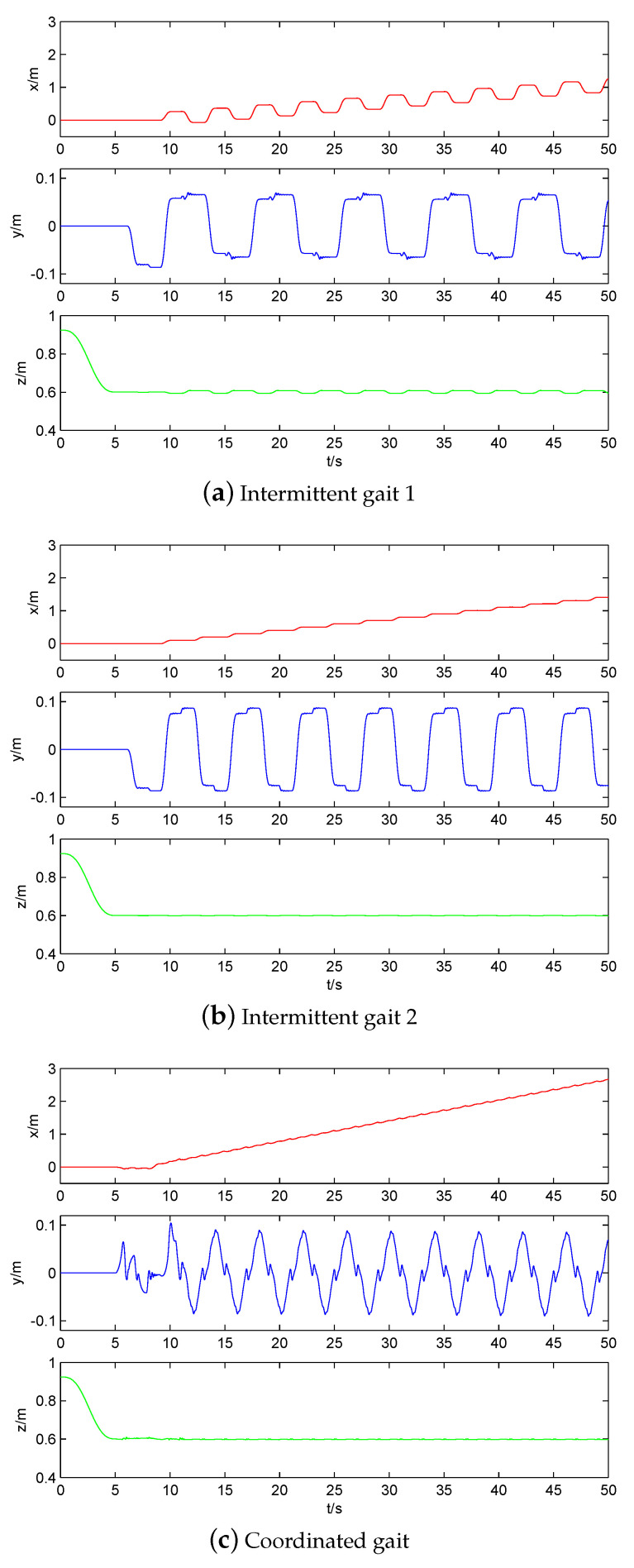
Comparison of CoM trajectory of three static gaits.

**Figure 11:**
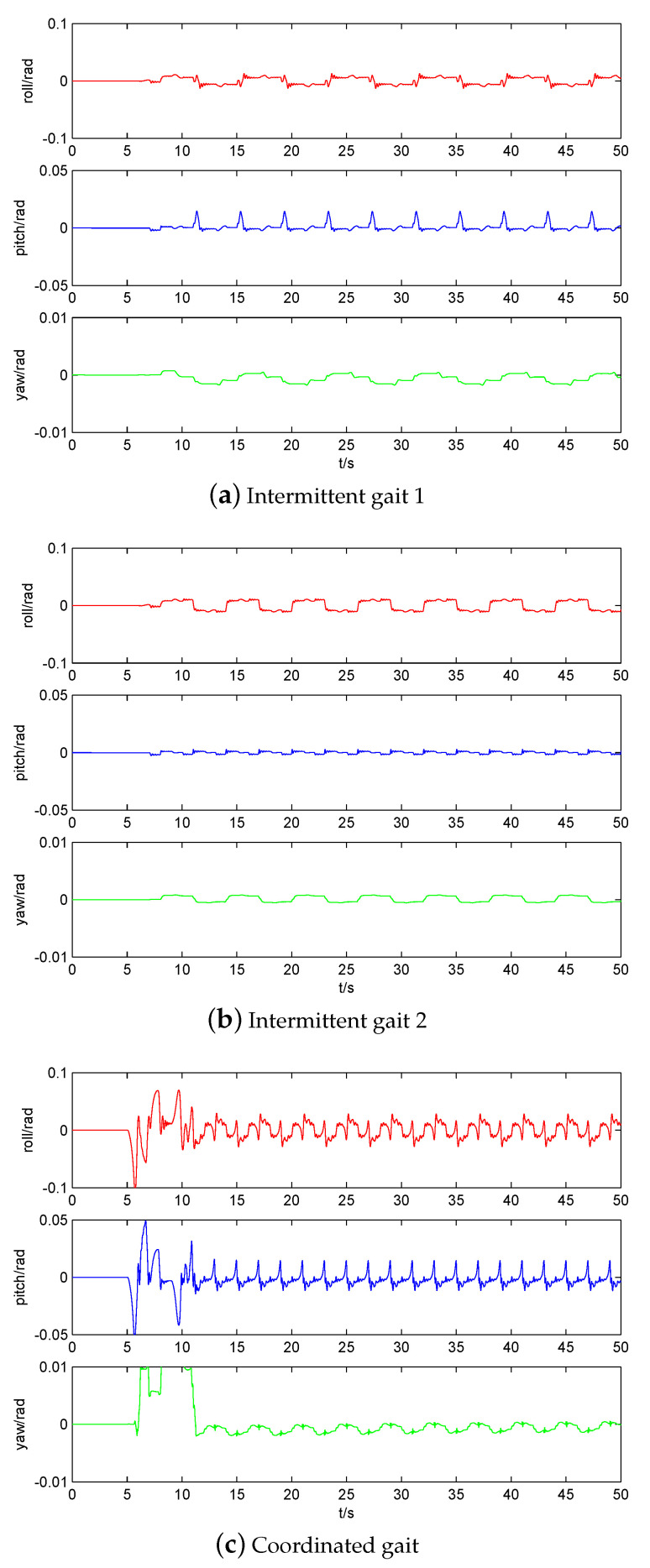
Comparison of attitude of three static gaits.

**Figure 12:**
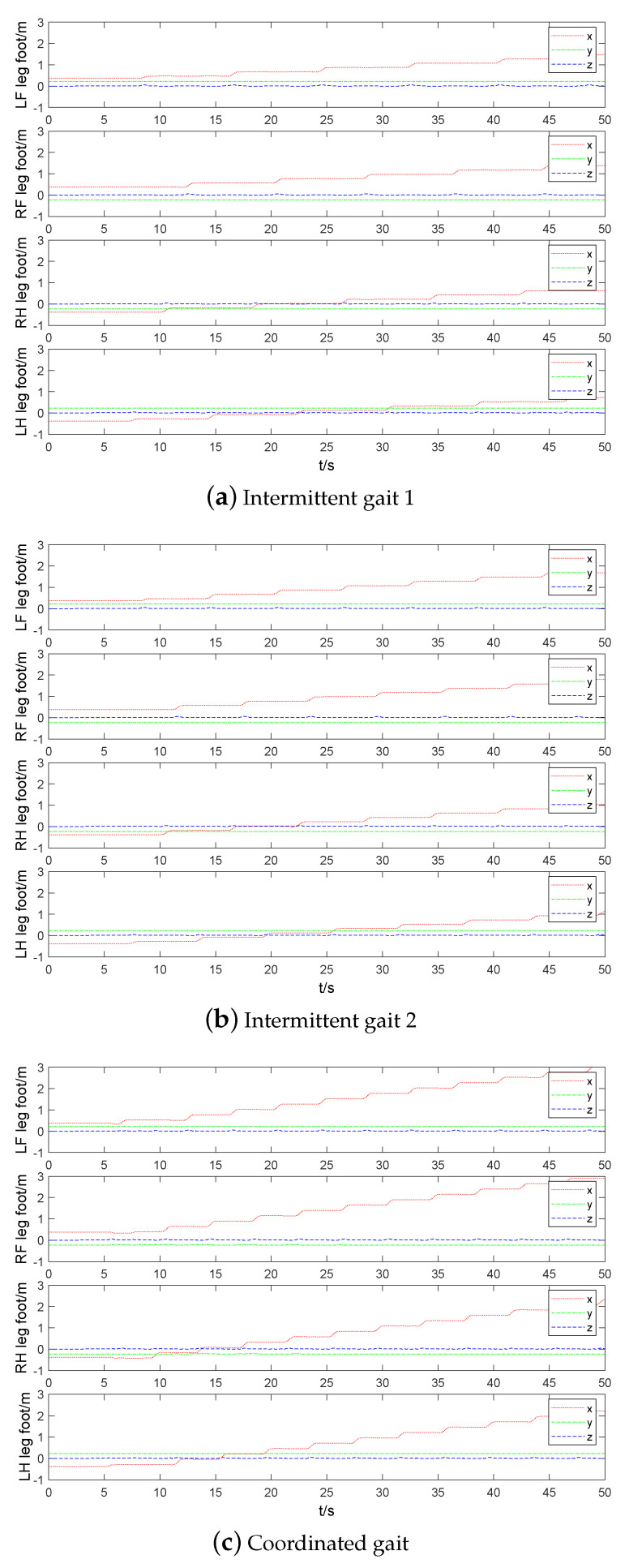
Comparison of foot end-effector of three static gaits.

**Figure 13:**
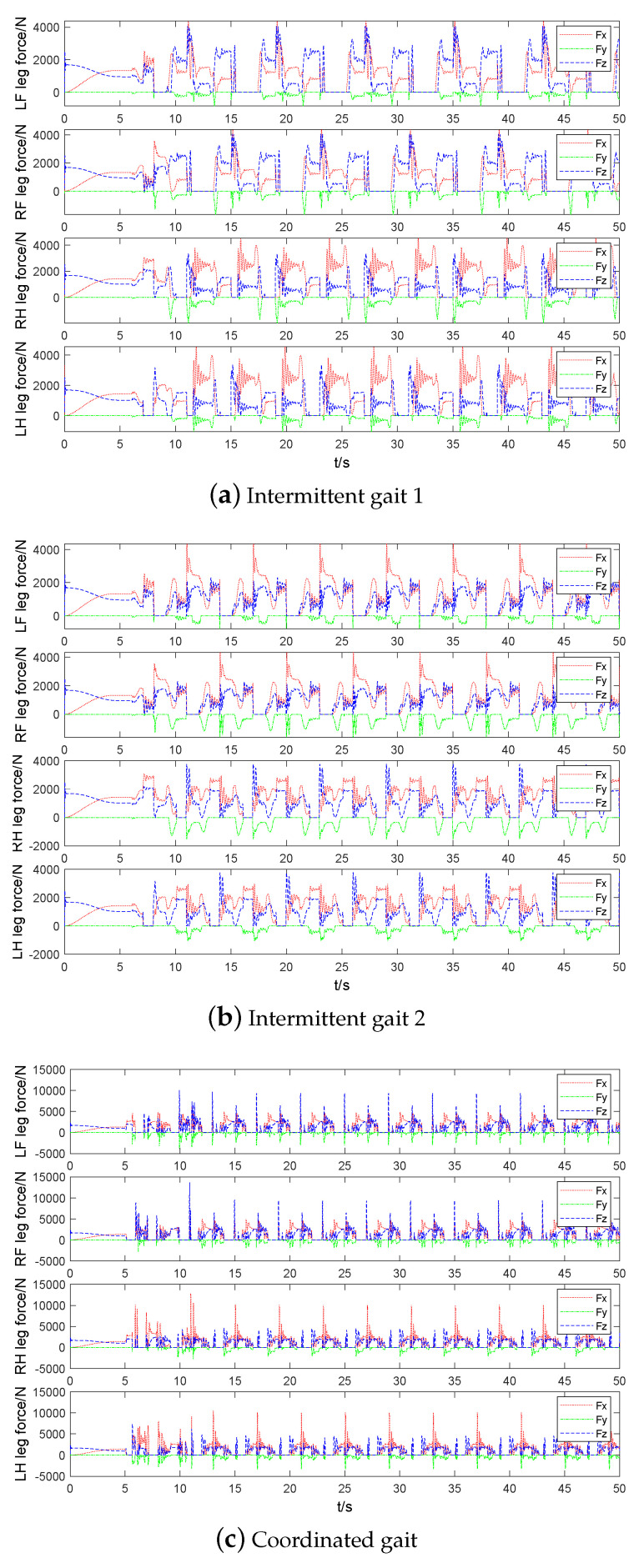
Comparison of foot contact force of three static gaits.

**Figure 14:**
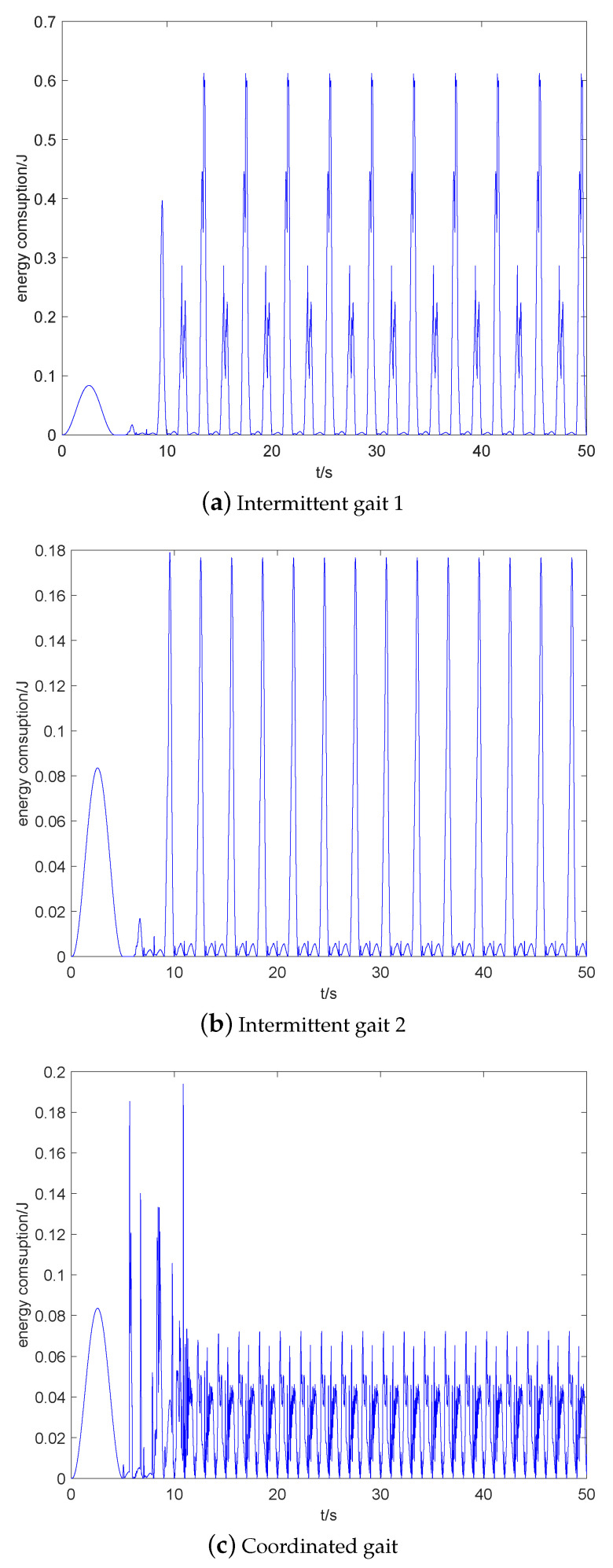
Comparison of energy consumption of three static gaits.

**Figure 15:**
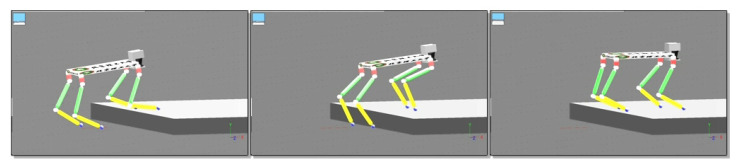
Simulation of quadruped robot walking on high platform with 0.2 m.

**Figure 16:**
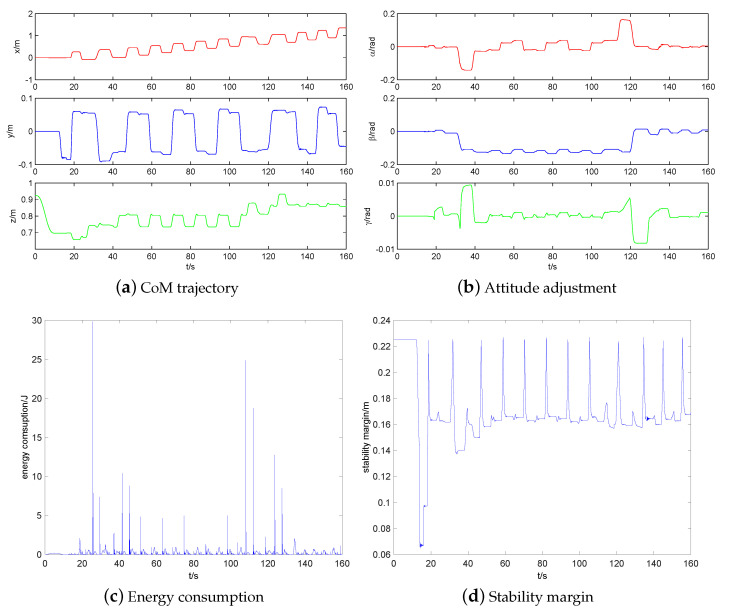
Simulation results of quadruped robot walking on a high platform.

**Figure 17:**
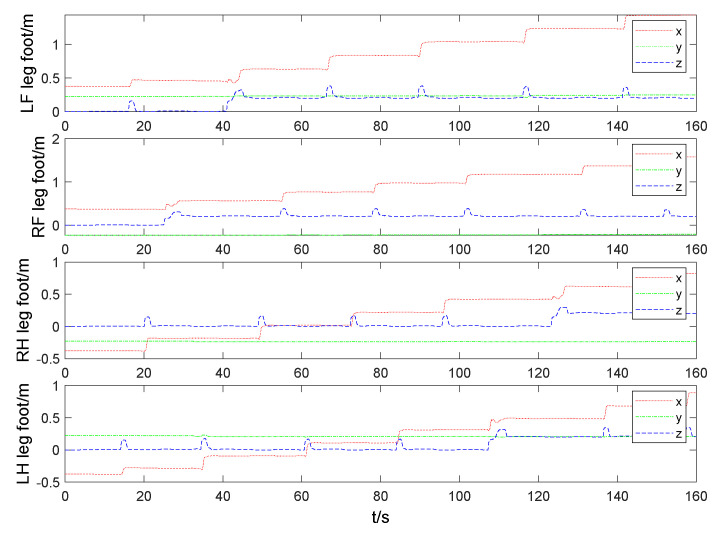
Comparison of four foot end-effector trajectories of quadruped robot walking on a high platform.

**Figure 18:**
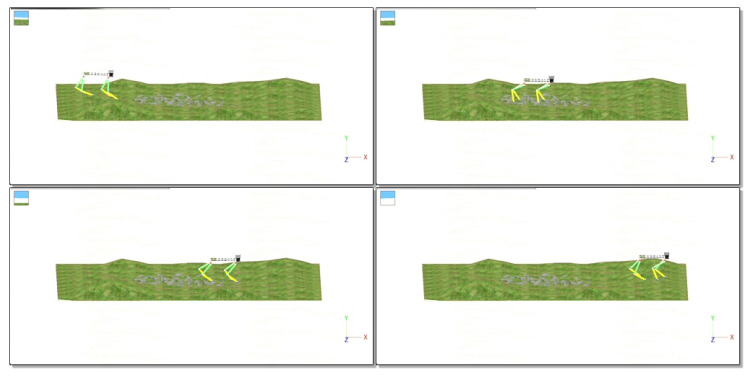
Simulation of the quadruped robot walking on uneven terrain.

**Figure 19:**
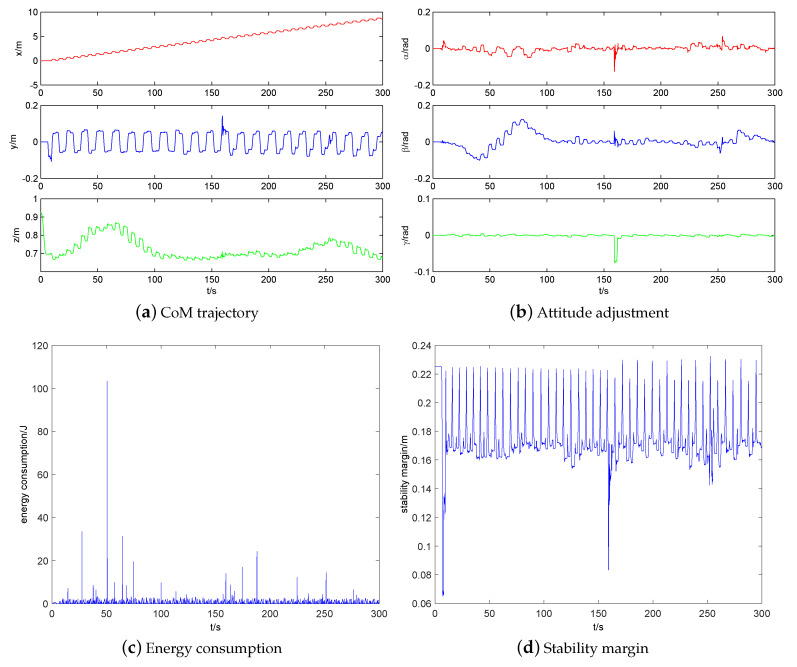
Simulation results of quadruped robot walking on uneven terrain.

Simulations are implemented by co-simulation between WEBOTS and MATLAB. WEBOTS is utilized to setup the dynamic model of quadruped robots. MATLAB is applied to designed the proposed controller. In MATLAB, ode4 (Runge–Kutta) solver is chosen and the fixed-step size is 0.001 s. In WEBOTS, the impact model is default. Simulations are employed to validate the proposed stability-guaranteed and high terrain adaptability static gait for a quadruped robot. The system and simulation parameters are shown in Table 1, where *S* is the step length. *H* is the step height. *T* is the time of one motion, such as one swing strike or one CoM movement. Simulations of quadruped robot walking with three static gaits (Figure 8, Figure 9, Figure 10, Figure 11, Figure 12, Figure 13 and Figure 14), walking on high platform (Figure 15, Figure 16 and Figure 17) and walking on rough terrain (Figure 18 and Figure 19) are analyzed as follows: Many simulation videos are shown as web links in the following Table 2.

### 5.1. Performance Index

Walking speed, CoT [51], and stability are three important performance indexes for quadruped robots. Walking speed is determined by gait planning in Equation (9). As for CoT, the work done by all actuators per walking distance unit is taken as CoT, and it yields

(25) $$\eta =\frac{W}{mgs}=\frac{\sum_{i=1}^{12}\left|T_{i}\theta_{i}\right|}{mgs}$$

where *s* is the walking distance, $\theta_{i}$ and $T_{i}$ are the joint angles and joint torques of robot, respectively.

Stability margin could be utilized to measure the static walking stability [52]. The stability margin is given as Equation (8).

### 5.2. Three Static Gaits

The simulations of quadruped robot walking with three static gaits: intermittent gait 1 (bottom), intermittent gait 2 (middle), and coordinated gait (upper) are shown in Figure 8.

Figure 9 shows the stability margin of three static gaits. It can be seen that stability margin of stable walking of intermittent gait 1, intermittent gait 2, and coordinated gait are 0.16∼0.225 m, 0.09∼0.225 m, 0∼0.125 m, respectively. Intermittent gait 1 has the highest stability margin, while coordinated gait has the lowest stability margin, even zero. At the moment, if the robot does not increase its stability margin or enter dynamic balance control, then the robot will fall over easily.

The CoM trajectory and attitude of three static gaits are compared in Figure 10 and Figure 11, respectively. Obviously, the CoM movement of coordinated gait is the smoothest in the direction of *x*-axis, and its walking distance is the farthest in the same 50 s time, but its stability margin is sacrificed. In the direction of the *y*-axis, in order to obtain a good stability margin, the left and right swing of three static gaits are almost the same. The fluctuation of coordinated gait at a zero crossing point is due to the stability margin having been close to zero. Note that the pendulum-like motion of robot upper body is to improve the walking stability of quadruped robot [29,30]. In the direction of the *z*-axis, the intermittent gait 1 has more oscillates because it makes more CoM shifts, including backward shifts. Figure 11 also shows that the increase of walking speed and excessive CoM movement will lead to the increase of attitude fluctuation.

The foot end-effector trajectory and contact force of three static gaits are compared in Figure 12 and Figure 13, respectively. Load factor refers to the ratio of single leg support time to the whole gait cycle. The smaller load factor, the faster walking speed. The load factors of three static gaits are 7/8,5/6,3/4, respectively. Thus, the coordination gait has the fastest walking speed. The swing leg time interval of coordination gait is short, which results in accelerating its swing leg frequency. Figure 13 shows that the contact force at the foot end-effector of the coordinated gait increases with the increase of walking speed, which causes more impact to the trunk so that trunk oscillations and the phenomenon of support legs leaving the ground occur. Four three-dimensional force sensors are installed at the foot end-effectors of quadruped robot and the *z*-axis follows the leg shank. Comparing the contact force of LF,RF and LH,RH, it can be seen that the main function of two front legs of robot is to prevent robot from falling forward, and the main function of two hind legs is to drive robot forward.

The energy consumptions of three static gaits are compared in Figure 14. The range of energy consumption for stable walking of three static gaits are 0∼0.6 J, 0∼0.18 J, 0∼0.07 J, respectively. It can be calculated that their total energy consumptions within 50 s are 4.21 kJ, 1.30 kJ, 1.46 kJ, respectively. Their CoTs are 4.87, 1.34, 0.79, respectively. It is easy to know that removing the redundant CoM movement can greatly reduce energy consumption, and the increase of walking speed does not mean the increase of energy consumption. Moreover, some part of the energy consumption of coordinated gait is lost to overcome the contact impact of ground.

### 5.3. Walk on High Platform

The simulation of the quadruped robot walking on a high platform with 0.2 m height is shown in Figure 15. The CoM trajectory, attitude adjustment, energy consumption, and stability margin of quadruped robot walking on a high platform are shown in Figure 16a–d, respectively. It can be seen that the horizontal velocity of CoM is stable. The horizontal swing changes very little. The vertical height changes with the height of high platform. The attitude angle changes in accordance with expectations in Equation (19). The yaw angle mutation is caused by the off-ground of the opposite supporting leg of swing leg when climbing up the high platform. The increase of energy consumption of robot occurs in the climbing up process, which conforms to the law of energy conservation. The stability margin is kept above 0.16 m, which shows that its stability is well guaranteed.

Especially due to the exploratory gait, the robot can sense the high platform, and then raise its CoM and foot to climb up the high platform. The four foot end-effector trajectories of quadruped robot walking on high platform are shown in Figure 17.

### 5.4. Walk on Uneven Terrain

The simulation of quadruped robot walking on uneven terrain is shown in Figure 18. The CoM trajectory, attitude adjustment, energy consumption, and stability margin of quadruped robot walking on uneven terrain are shown in Figure 19a–d, respectively. The simulation results are consistent with those of quadruped robot walking on a high platform. Note that the reason of the vibration near 160 s is that the shank of a swing leg of the robot touches a rock. Because the front and hind legs of the robot are all elbow style configuration, the walking up ability of the robot is strong while the walking down ability of the robot is weak. The shank of the swing leg of robot will touch the ground easily during the descending process, which will cause unstable walking. Especially when the robot walks down stairs, the shank of the swing leg of the robot will be impacted by the stair edge. As thus, the robot will fall over while going down the stairs easily.

Further research is implemented to calculate the CoT of quadruped robot walking on different terrains by using intermittent gait 1, as shown in Table 2. The CoT of quadruped robot walking on even terrain, walking on slopes without impedance control, walking on slopes with impedance control, walking on stairs with 0.01 m height, walking on high platform with 0.02 m height, and walking on uneven terrain are 4.87,5.52,5.33,6.42,6.45, respectively. It can be found that the CoT of quadruped robot walking on even terrain is the lowest because other gaits have to overcome gravity to do work. In addition, the introduction of impedance control can reduce the contact impact and CoT to some extent. Moreover, the stability margin is less affected by terrain, which is mainly determined by the chosen gait itself (intermittent gait 1).

### 5.5. Performance Analysis

Stability margin is taken in the first place. In addition, three static gaits are then chosen. The three static gaits can eliminate unnecessary motions, consume less energy, reduce gait cycle, and are more bionic. Among the 24 walk gait combinations in [17], the three static gaits have the best comprehensive performance in walking speed, CoT, and stability.

**Figure 20:**
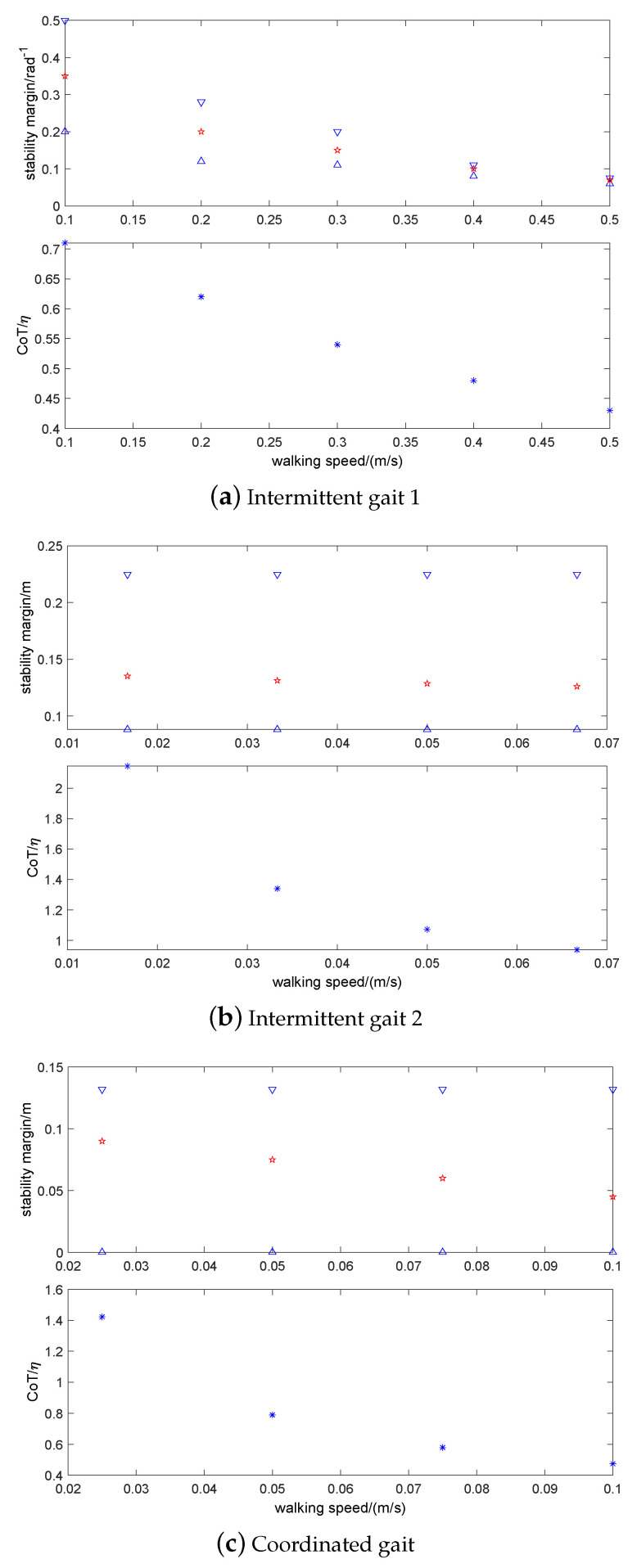
Relationship among walking speed, CoT and stability of three static gaits (∇,Δ&* are upper and lower limits and mean values, respectively).

In order to further analyze the relationship among walking speed, CoT, and stability of static gait, three static gaits with four different speeds are compared by changing the gait and gait cycle, as shown in Figure 20.

It can be seen that the increase of walking speed among three static gaits directly leads to the decrease of stability margin, but the CoT is reduced as well. In the same static gait, the increase of walking speed can also effectively reduce CoT, which results in a certain degree of the attenuation effect on the stability margin. Therefore, the factors that need to be considered in static gait selection contain terrain complexity, stability, walking speed, and CoT.

## 6. Conclusions

This paper proposed a stability-guaranteed and high terrain adaptability static gait for quadruped robots. The main contributions can be concluded as follows:

- Three stability-guaranteed static gaits: intermittent gait 1&2 and coordinated gait, are investigated, and a novel exploratory gait planning on uneven terrain with touch sensing is proposed to improve the terrain adaptability.
- A position/force based active compliance controller is introduced into static gaits to improve compliant behavior and energy efficiency of quadruped robots.
- A novel attitude-position adjustment strategy with terrain estimation is proposed to improve the walking stability.
- Proposed methods are validated by simulations, and the relationships among four factors for static gait selection: terrain complexity, stability, walking speed, and CoT are discussed.

Future works focus on multi-body dynamics, anti-disturbance, AI adaptivity, etc.

## Figures and Tables

**Table 1:** System and simulation parameters.

Parameters	Value
$l_{1},l_{2},l_{3}$	0.08 m, 0.41 m, 0.43 m
$\theta_{1},\theta_{2},\theta_{3}$	$-33^{\circ }∼25^{\circ },7^{\circ }∼88^{\circ },46^{\circ }∼133^{\circ }$
$L,W,H_{B}$ (Body height)	1.1 m, 0.57 m, 1.08 m
$m_{1},m_{2},m_{3},m_{B}$	1 kg, 2 kg, 2 kg, 50 kg
Walking height	0.45 m ∼ 0.83 m
Mass, Load	60 kg, 300 kg
S,H,T	0.2 m, 0.05 m, 1 s

**Table 2:** Simulation video links.

Simulations	Links
There static gait on even terrain	https://youtu.be/V0yh1IJxOmk
high platform with 0.2 m height	https://youtu.be/FM_4M2dytno
uneven terrain	https://youtu.be/-fPBTk_APX8
stairs with 0.1 m height	https://youtu.be/dLgKdVpJlo0
0.2 rad slope with impedance control	https://youtu.be/nL815_TEYh4
0.2 rad slope without impedance control	https://youtu.be/v1mqAgksb4Q
